# Vascular and Cardiac Impairments in Rats Inhaling Ozone and Diesel Exhaust Particles

**DOI:** 10.1289/ehp.1002386

**Published:** 2010-10-27

**Authors:** Urmila P. Kodavanti, Ronald Thomas, Allen D. Ledbetter, Mette C. Schladweiler, Jonathan H. Shannahan, J. Grace Wallenborn, Amie K. Lund, Matthew J. Campen, Elizabeth O. Butler, Reddy R. Gottipolu, Abraham Nyska, Judy E. Richards, Deborah Andrews, Richard H. Jaskot, John McKee, Sainath R. Kotha, Rishi B. Patel, Narasimham L. Parinandi

**Affiliations:** 1 Environmental Public Health Division, National Health and Environmental Effects Research Laboratory, Office of Research and Development, U.S. Environmental Protection Agency. Research Triangle Park, North Carolina, USA; 2 Curriculum in Toxicology and; 3 School of Public Health, University of North Carolina, Chapel Hill, North Carolina, USA; 4 Toxicology Division, Lovelace Respiratory Research Institute, Albuquerque, New Mexico, USA; 5 College of Pharmacy, University of New Mexico, Albuquerque, New Mexico, USA; 6 Davis Heart and Lung Research Institute, The Ohio State University College of Medicine, Columbus, Ohio, USA; 7 Tel Aviv University, Tel Aviv, Israel; 8 Research Core Units, National Health and Environmental Effects Research Laboratory, Office of Research and Development, U.S. Environmental Protection Agency, Research Triangle Park, North Carolina, USA

**Keywords:** air pollution, aorta, cardiovascular, diesel exhaust particles, inhalation, LOX-1, ozone, vascular

## Abstract

**Background:**

Mechanisms of cardiovascular injuries from exposure to gas and particulate air pollutants are unknown.

**Objective:**

We sought to determine whether episodic exposure of rats to ozone or diesel exhaust particles (DEP) causes differential cardiovascular impairments that are exacerbated by ozone plus DEP.

**Methods and results:**

Male Wistar Kyoto rats (10–12 weeks of age) were exposed to air, ozone (0.4 ppm), DEP (2.1 mg/m^3^), or ozone (0.38 ppm) + DEP (2.2 mg/m^3^) for 5 hr/day, 1 day/week for 16 weeks, or to air, ozone (0.51 or 1.0 ppm), or DEP (1.9 mg/m^3^) for 5 hr/day for 2 days. At the end of each exposure period, we examined pulmonary and cardiovascular biomarkers of injury. In the 16-week study, we observed mild pulmonary pathology in the ozone, DEP, and ozone + DEP exposure groups, a slight decrease in circulating lymphocytes in the ozone and DEP groups, and decreased platelets in the DEP group. After 16 weeks of exposure, mRNA biomarkers of oxidative stress (hemeoxygenase-1), thrombosis (tissue factor, plasminogen activator inhibitor-1, tissue plasminogen activator, and von Willebrand factor), vasoconstriction (endothelin-1, endothelin receptors A and B, endothelial NO synthase) and proteolysis [matrix metalloprotease (*MMP*)-*2*, *MMP-3*, and tissue inhibitor of matrix metalloprotease-2] were increased by DEP and/or ozone in the aorta, but not in the heart. Aortic *LOX-1* (lectin-like oxidized low-density lipoprotein receptor-1) mRNA and protein increased after ozone exposure, and LOX-1 protein increased after exposure to ozone + DEP. *RAGE* (receptor for advanced glycation end products) mRNA increased in the ozone + DEP group. Exposure to ozone or DEP depleted cardiac mitochondrial phospholipid fatty acids (DEP > ozone). The combined effect of ozone and DEP exposure was less pronounced than exposure to either pollutant alone. Exposure to ozone or DEP for 2 days (acute) caused mild changes in the aorta.

**Conclusions:**

In animals exposed to ozone or DEP alone for 16 weeks, we observed elevated biomarkers of vascular impairments in the aorta, with the loss of phospholipid fatty acids in myocardial mitochondria. We conclude that there is a possible role of oxidized lipids and protein through *LOX-1* and/or *RAGE* signaling.

Cardiac physiological impairments have been noted after ozone exposure in rodents (Uchiyama et al. 1986; Watkinson et al. 2001). Studies have shown that ambient particulate matter (PM) and diesel exhaust particles (DEP) cause decreased heart rate variability (Chuang et al. 2007) and impair endothelial function in humans (Peretz et al. 2008). Moreover, PM exposure has been associated with myocardial ischemia (Zhang et al. 2009), microvascular thrombosis (Lucking et al. 2008), atherosclerosis (Kunzli et al. 2010), and diabetes in humans (Kramer et al. 2010). Although a few animal experiments with PM and ozone provide supportive evidence for human associations (Chuang et al. 2009; Sun et al. 2009), the molecular mechanism(s) for cardiopulmonary interactions are unknown.

One of the mechanisms proposed for PM-induced cardiovascular effects involves systemic translocation of the leachable components, such as metals, causing direct cardiovascular impairment (Wallenborn et al. 2008, 2009); however, it is not known how vascular effects are caused by pollutants that do not translocate systemically. The release of pulmonary cytokines and vasoactive mediators in the circulation has been proposed to cause systemic endothelial changes. A few studies have shown temporal increases in the circulating cytokines after PM exposure (Swiston et al. 2008; van Eeden et al. 2005); however, in many cases no such increases have been reported (Cassee et al. 2005; Gottipolu et al. 2009; Kodavanti et al. 2008; Wilson et al. 2010). Thus, some cardiovascular effects of PM might be mediated by yet unexplained mechanisms.

Both diabetes and atherosclerosis have been linked to PM exposure in epidemiological studies and in limited animal studies (Pereira Filho et al. 2008; Sun et al. 2009). The primary cause of diabetic vascular complications involves activation of endothelial receptors for advanced glycation end products (*RAGE*), which recognizes circulating oxidatively modified proteins (Yan et al. 2009). Similarly, vascular complications of atherosclerosis are mediated by lectin-like oxidized low-density lipoprotein-1 (*LOX-1*) receptors that bind oxidatively modified lipids and stimulate downstream signaling involving nuclear factor kappa B (*NF*κ*B*) (Navarra et al. 2009). It is likely that inhaled pollutants cause oxidation of key proteins and lipids, and their presence in circulation promotes *RAGE* and *LOX-1* signaling and downstream vascular oxidative stress, inflammation, vasoconstriction, protease/antiprotease imbalance, and thrombosis.

We have shown cardiac mitochondrial oxidative stress and suppression of genes for antioxidant compensatory mechanisms in the absence of increases in biomarkers associated with cardiac inflammation and thrombosis in rats inhaling diesel exhaust and other PM (Gottipolu et al. 2009; Kodavanti et al. 2008). Cellular mitochondrial oxidative stress has been shown to cause membrane phospholipid hydrolysis, generation of bioactive lipid signaling mediators, and formation of reactive intermediates of polyunsaturated fatty acids (PUFA) (Kagan et al. 2009). However, it is not known if myocardial mitochondrial phospholipids are altered after exposure to ozone or DEP.

We hypothesized that both ozone and DEP exposure would cause aortic changes consistent with oxidative stress, inflammation, thrombosis, vasoconstriction, and proteolysis involving *RAGE, LOX-1*, and cardiac membrane phospholipid fatty acid oxidation in healthy rats, and that exposure to ozone plus DEP would lead to additive or synergistic interactions.

## Materials and Methods

### Animals

We purchased healthy male Wistar Kyoto rats, 10–12 weeks of age, from Charles River Laboratories Inc. (Raleigh, NC, USA). Rats were acclimatized (21 ± 1°C, 50 ± 5% relative humidity, and 12-hr light/dark cycle) for 1 week in an animal facility approved by the Association for Assessment and Accreditation of Laboratory Animal Care International. All animals received standard Purina rat chow (Purina Mills, Brentwood, MO, USA) and water *ad libitum*. The use of animals in this study was approved by the Animal Care and Use Committee of the National Health and Environmental Effects Research Laboratory, U.S. Environmental Protection Agency (EPA). The animals were treated humanely and with regard for alleviation of suffering.

### Ozone and DEP aerosol generation

A bulk DEP sample was collected using a bag house collection devise during operation of a stationary 30-kW Deutz engine as described previously (Shinyashiki et al. 2009; Stevens et al. 2009). DEP particles were aerosolized using a string generator system (Ledbetter et al. 1998) for distribution to a 24-port flow-by nose-only inhalation chamber [see Supplemental Material, “Methods” (doi:10.1289/ehp.1002386)]. In the 16-week study, ozone was generated using a gas phase titration diluter. The ozone concentration was monitored continuously using a photometric ozone analyzer (Model 400; Advanced Pollution Instruments, San Diego, CA, USA). For acute exposures, ozone was generated from oxygen using an OREC silent arc discharge ozone generator (Osmonics, Phoenix, AZ, USA) [see Supplemental Material, “Methods” (doi:10.1289/ehp.1002386)].

### Animal exposure

For the episodic study, rats (*n* = 20/group) were exposed for 5 hr/day, 1 day/week for 16 weeks, to either ozone or DEP or to a combination of ozone + DEP, based on our previous studies (Kodavanti et al. 2008). The desired chamber concentrations were 0.5 ppm ozone and 2.0 mg/m^3^ DEP. To determine whether effects observed after 16 weeks of episodic exposure were due to an effect of the last exposure, we also performed an acute study in which rats were exposed to air, ozone (0.5 or 1.0 ppm), or DEP (2.0 mg/m^3^) for 5 hr/day for 2 consecutive days [see Supplemental Material, “Methods” (doi:10.1289/ehp.1002386)].

### Monitoring of breathing parameters

We used whole-body plethysmography to determine the effect, if any, of DEP, ozone, or ozone + DEP on the respiratory system each week prior to and after exposure in the 16-week study [see Supplemental Material, “Methods” (doi:10.1289/ehp.1002386)]. Automated breath-by-breath analyses were performed using a rejection algorithm described previously (Kodavanti et al. 2005).

### Necropsy, sample collection, and bronchoalveolar lavage fluid (BALF) analysis

Two days after 16-week episodic exposure (16-week study) or 1 day after 2 consecutive days of exposure (acute study), rats (*n* = 6/group) were anesthetized with sodium pentobarbital (50–100 mg/kg, intraperitoneal injection). Blood was collected from the abdominal aorta into collection tubes containing EDTA (for complete blood count), citrate (for plasma protein analysis), or no anticoagulant (serum for metabolic markers). The heart was weighed and cut into two mid-longitudinal halves. One half was fixed in 10% neutral formalin, and the left ventricle portions of the second half were snap-frozen in liquid nitrogen and retained at −80^o^C.

The right lung was lavaged with Ca^++^/Mg^++^-free phosphate-buffered saline (pH 7.4) as described previously (Gottipolu et al. 2009). Lavaged right lung lobes were quick-frozen in liquid nitrogen for later RNA extraction. The left lung was tracheally fixed with neutral formalin for later histological evaluation. The thoracic aorta was cut into three pieces; a portion of the aorta at the origin was fixed in formalin, and the rest was cut into two pieces and stored at −80°C for later analysis. A portion of spleen was also fixed in formalin for histological evaluation. BALF was processed for total cell counts and lung injury markers (protein, albumin, and lactate dehydrogenase activity) as previously reported (Gottipolu et al. 2009).

### Blood chemistry and cytology

In both 16-week and acute studies, aliquots of blood collected into tubes containing EDTA, citrate, or no coagulants (for serum) were used for various analyses. Complete blood counts were performed using a Beckman-Coulter AcT blood analyzer (Beckman-Coulter Inc., Fullerton, CA, USA). Fibrinogen, adiponectin, and metabolic markers were analyzed in the plasma or serum samples [see Supplemental Material, “Methods” (doi:10.1289/ehp.1002386)].

### Tissue processing for histology

In the 16-week study, 2–3 μm sections of left lung, heart, thoracic aorta, and spleen were stained with hematoxylin and eosin for examination by light microscopy [see Supplemental Material, “Methods” (doi:10.1289/ehp.1002386)]. All lesions observed were graded on a four-point scale (1, minimal; 2, mild; 3, moderate; and 4, marked), based on published criteria (Nyska et al. 2005).

### RNA isolation and real-time PCR

Total lung, heart, and thoracic aorta RNA for the 16-week study and lung and aorta RNA for the acute study were isolated using RNeasy mini kits (Qiagen, Valencia, CA, USA). We performed one-step real-time reverse-transcription polymerase chain reaction (RT-PCR) with the SuperScript III Platinum One-Step Quantitative RT-PCR kit from Invitrogen (Carlsbad, CA, USA); and target-specific primers using the ABI Prism 7900 HT sequence detection system (Applied Biosystems, Foster City, CA, USA) [see Supplemental Material, “Methods” (doi:10.1289/ehp.1002386)].

### Aorta LOX-1 and RAGE protein analysis

In the 16-week study, extracts from thoracic aorta were analyzed for protein using a Bradford reagent kit (Biorad, Hercules, CA, USA) and were subjected to SDS-PAGE electrophoresis. Membranes were incubated with rabbit polyclonal anti-mouse LOX-1 (Abcam, Cambridge, MA, USA) or anti-rat β-actin and developed using chemiluminescence. Densitometry was performed using Image J software (National Institutes of Health 2010). For RAGE, we used anti-rat (c20) polyclonal goat antibody (Santa Cruz Biotechnology, Inc., Santa Cruz, CA, USA) [see Supplemental Material, “Methods” (doi:10.1289/ehp.1002386)].

### Cardiac mitochondria, lipid extraction, and fatty acid analysis

For the 16-week study, a separate group of rats was anesthetized as described above to prepare cardiac mitochondria fractions (*n* = six rats/group). We used a small portion of the left ventricle for isolation of mitochondria (Gottipolu et al. 2009). Lipid extraction and analysis of fatty acids were performed as described previously (Parinandi et al. 1990a, 1990b). Briefly, lipids were extracted, and phospholipids were methylated by alkaline methanolysis to prepare fatty acid methyl esters. The resulting fatty acid methyl esters were analyzed by gas chromatography-mass spectrometry (GC-MS) using the Shimadzu QP2010 GC-MS (Shimadzu Scientific Instruments, Columbia, MD, USA) equipped with Restek column. Fatty acids were normalized to milligrams of mitochondrial protein.

### Statistical analysis

Data were analyzed by one-way analysis of variance (ANOVA) using SigmaStat, version 3.5 (Systat Software, Inc., Point Richmond, CA, USA). This statistical program automatically selected the most appropriate test with each biomarker depending on the distribution of values within a given data set. In some comparisons, we used Kruskal-Wallis one-way ANOVA on ranks. Group comparisons were made using the Holm-Sidak, Tukey’s, or Dunn’s method. The Mann-Whitney rank sum test was used in individual group comparisons for some biomarkers. We considered a *p*-value of 0.05 to be statistically significant.

## Results

### Ozone and DEP exposure characteristics

The daily mean (± SD) concentrations of ozone and DEP for all three exposure groups are given in Supplemental Material, Table 1 (doi:10.1289/ehp.1002386). The actual mean (± SD) ozone concentrations achieved in the 16-week study for ozone only and for ozone + DEP chambers were 0.403 ± 0.051 and 0.382 ± 0.064 ppm, respectively, and the mean (± SD) DEP concentrations for DEP only and for ozone + DEP were 2.14 ± 0.66 and 2.19 ± 1.10 mg/m^3^, respectively. The mass medial aerodynamic diameter (MMAD) was 1.20 μm [geometric standard deviation (GSD), 2.67] [see Supplemental Material, “Results” (doi:10.1289/ehp.1002386)]. In the acute study, the mean ozone concentrations achieved for the 0.5 ppm and 1.0 ppm chambers were 0.51 ppm and 1.00 ppm, respectively, and the mean DEP concentration was 1.89 mg/m^3^. The MMAD was 0.86 μm (GSD, 2.42). The composition of the DEP bulk particle sample has been described previously (Shinyashiki et al. 2009; Stevens et al. 2009).

### Body weights and breathing parameters after episodic (16-week) exposure

**Figure 1:**
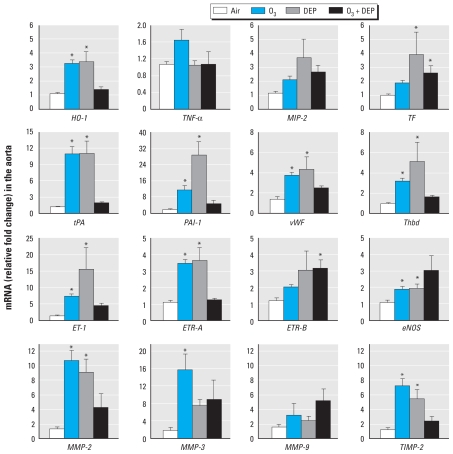
Episodic 16-week exposure to air, ozone (O_3_; 0.5 ppm), DEP (2 mg/m^3^), or O_3_ + DEP induces mRNA expression for biomarkers of oxidative stress, thrombosis, vasoconstriction, and proteolytic balance in the aorta. mRNA expression was analyzed using real-time PCR. Values shown are mean ± SEM of six animals. For *MMP-2, MMP-3, and TIMP-2*, one-way ANOVA followed by Holm–Sidak test was used; for all other markers, Kruskal–Wallis one-way ANOVA on ranks was followed by Dunn’s test. *p ≤ 0.05 relative to air controls.

As determined on the day after exposure, the average weight losses for the air, ozone, DEP, and ozone + DEP groups were −3 g, −9 g, −4 g, and −8 g, respectively. We observed no significant effect of time × exposure; however, we did note some significant time-related and ozone exposure–related differences (*p* ≤ 0.05). [See Supplemental Material, “Results” and Figure 1 (doi:10.1289/ehp.1002386)].

**Figure 2:**
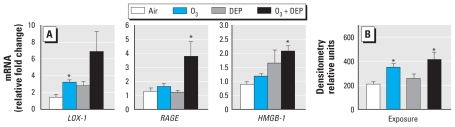
Expression of *LOX-1*, *RAGE*, and *HMGB-1* mRNA (*A*) and LOX-1 protein (*B*) in the aorta of rats after episodic exposure to air, ozone (O_3_; 0.5 ppm), DEP (2 mg/m^3^), or O_3_ + DEP. mRNA expression was analyzed using real-time PCR, and protein extracts were analyzed for LOX-1 using Western blotting. Values shown are mean ± SEM of six animals. The Mann-Whitney rank sum test was used for mRNA data; for LOX-1 protein analysis, Kruskal–Wallis one-way ANOVA on ranks was followed by post hoc comparison using Dunn’s test. *p ≤ 0.05 relative to air controls.

We observed no significant changes in any of the breathing parameters [breathing frequency, tidal volume, minute volume, inspiratory time, expiratory time, or PenH (enhanced pause)] after DEP exposure (data not shown). However, all ozone-exposed rats (ozone, ozone + DEP) exhibited increased PenH [see Supplemental Material, Figure 2, (doi:10.1289/ehp.1002386)]. Increased PenH provides an index of labored breathing in some types of airway injuries and is computed by integration of breathing parameters and the time spans of inhalation and exhalation. The effect of ozone 1 day after the first week of exposure was greater than the effects seen during subsequent weeks of exposure (3–16 weeks), suggesting partial adaptation [see Supplemental Material, “Results” (doi:10.1289/ehp.1002386)].

### Pulmonary and systemic alterations after 16-week exposure

**Figure 3:**
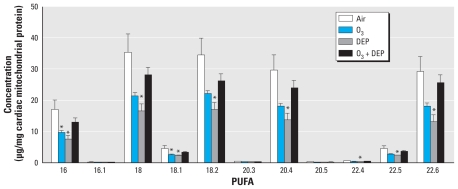
Depletion of cardiac mitochondrial PUFA after episodic exposure to air, ozone (O_3_; 0.5 ppm), DEP (2 mg/m^3^), or O_3_ + DEP in rats. PUFA were analyzed by GC-MS. Values shown are mean ± SEM of six animals. Fatty acid designations are as follows: C16:0, palmitic acid; C16:1, palmitoleic acid; C18:0, stearic acid; C18:1, oleic acid; C18:2, linoleic acid; C20:3, dihomo-g-linolenic acid; C20:4, arachidonic acid; C20:5, eicosapentaenoic acid; C22:4, docosatetraenoic acid; C22:5, doecosapentaenoic acid; and C22:6, docosahexaenoic acid. Kruskal–Wallis one-way ANOVA on ranks followed by Dunn’s test was employed for fatty acid 20.4; one-way ANOVA followed by Holm–Sidak test was used for all other markers. *p ≤ 0.05 relative to air controls.

We assessed several pulmonary and systemic biomarkers in order to understand their potential contributions in cardiac and vascular toxicity. No significant changes were observed in total cells or alveolar macrophages; however, we noted a small but significant increase in neutrophils in BALF after DEP exposure [see Supplemental Material, Figure 3A (doi:10.1289/ehp.1002386)]. This effect of DEP was not evident in rats exposed to ozone + DEP. BALF protein, albumin, and lactate dehydrogenase activity were not increased by any of the exposure regimen (see Supplemental Material, Figure 3B). Rather, lactate dehydrogenase activity was reduced in rats exposed to ozone.

Circulating hemoglobin levels were slightly but significantly increased by ozone or DEP but not by ozone + DEP, whereas platelets were decreased in DEP-exposed rats [see Supplemental Material, “Results” and Table 2 (doi:10.1289/ehp.1002386)]. Circulating lymphocytes, when calculated as the percentage of total white blood cells, decreased with all exposures (*p* ≤ 0.05); however, this trend did not reach significance when the data were expressed as cells per milliliter of blood. Plasma fibrinogen was slightly decreased by DEP. We observed no other notable exposure-related changes in clinical markers of metabolic disorder and thrombosis (see Supplemental Material, “Results” and Table 2).

### Pulmonary, cardiac, and aortic histological changes after 16-week exposure

The number of animals affected and mean lung pathology severity score per animal are shown in Supplemental Material, Table 3 (doi:10.1289/ehp.1002386). All exposures caused mild histological alterations in the lung. Macrophages contained numerous dark DEP granules of various sizes in the alveoli and the lymphoid aggregates along major airways in DEP and ozone + DEP–exposed rats (see Supplemental Material, Figure 4). We noted mild thickening of terminal bronchioles, alveolar ducts, and adjacent alveoli, with a small degree of interstitial inflammation, in ozone and ozone + DEP–exposed rats. No exposure-related changes were observed in the heart, spleen, or aorta (see Supplemental Material, Table 3).

### Expression of mRNA biomarkers in the lung, heart, and aorta after 16-week exposure

To determine molecular alterations within the lung, heart, and the aorta in parallel, we analyzed a number of biomarkers at the mRNA level. The biomarkers were selected to reflect oxidative stress [hemeoxygenase-1 (*HO-1*)], proteolytic activity [matrix metalloprotease (*MMP*)-*2*, *MMP-3, MMP-9*, and tissue inhibitor of matrix metalloprotease-2 (*TIMP-2*)], inflammation [macrophage inflammatory protein-2 (*MIP-2*) and tumor necrosis factor-α (*TNF-*α)], prothrombotic changes [tissue factor (*TF*), tissue plasminogen activator (*tPA*), plasminogen activator inhibitor-1 (*PAI-1*), von Willebrand factor (*vWF*), thrombomodulin (*Thbd*)], vascular contractility alterations [endothelin-1 (*ET-1*), endothelin receptor-A (*ETR-A*), endothelin receptor-B (*ETR-B*), endothelial NO synthase (*eNOS*), angiotensin-II, atrial natriuretic peptide (*ANP*), and brain natriuretic peptide (*BNP*)], and signaling through the receptors that recognize circulating protein oxidation end products (*RAGE*) and *LOX-1*. Surprisingly, we saw no remarkable effects on any of the biomarkers in the left ventricular tissue with any of the exposures [see Supplemental Material, Table 4 (doi:10.1289/ehp.1002386)]. Small but significant increases in lung *TNF*-α were present after exposure to ozone or DEP, and *MIP-2* was increased after DEP exposure. Similarly, three biomarkers were slightly increased in rats after exposure: *tPA* after ozone or DEP, *PAI-1* after DEP, and *TF* after ozone + DEP (see Supplemental Material, Table 4).

Interestingly, we observed marked up-regulation in biomarkers in the aorta (Figure 1). *HO-1* mRNA was increased in the aorta of rats exposed to ozone or DEP, but not ozone + DEP. The expression of *MIP-2* or *TNF-*α was not altered by any of the exposures. *TF* increased in DEP and ozone + DEP–exposed rats. Ozone or DEP alone, but not ozone + DEP, increased *tPA*, *PAI-1*, *vWf*, and also *Thbd* (Figure 1). Data suggest that both ozone and DEP individually induced prothrombotic changes in the aorta.

Expression of *ET-1* and *ETR-A* was enhanced after exposure to ozone or DEP, but not ozone + DEP (Figure 1), whereas *ETR-B* increased only with ozone + DEP. Angiotensin-II, *ANP*, and *BNP* mRNA were variable in all exposure groups including controls, and no consistent differences could be ascertained (data not shown). The expression of *eNOS* increased slightly but significantly in ozone- or DEP-exposed rats; however, this trend was not significant in the case of ozone + DEP.

*MMP-2* was up-regulated several-fold by ozone or DEP in the aorta (Figure 1). *MMP-3* increased significantly with ozone, but the trends for DEP and ozone + DEP were not significant. *MMP-9* was not up-regulated by any exposures; however, *TIMP-2* expression was increased by both ozone and DEP. *TIMP-2* expression in ozone + DEP–exposed rats was not different from controls (Figure 1).

*LOX-1* mRNA tended to increase after exposure to ozone and ozone + DEP; however, only the ozone group reached the level of significance (*p* < 0.05). *RAGE* expression increased only in ozone + DEP–exposed rats. High-mobility group box-1 (*HMGB-1*), shown to be involved in chronic inflammation, was up-regulated in aortas from rats exposed to ozone + DEP (Figure 2A).

There was an increase in LOX-1 protein in rats exposed to ozone and ozone + DEP. Although LOX-1 protein was increased by DEP exposure, it was not significant (Figure 2B). We observed no consistent changes in RAGE protein with any exposures (data not shown).

### Loss of cardiac mitochondrial phospholipid fatty acids after 16-week exposure

Both ozone and DEP exposures decreased saturated, monounsaturated, and PUFA in myocardial mitochondria (Figure 3). In DEP-exposed rats, fatty acids decreased in every measureable case, whereas with ozone, the decrease was significant with only two fatty acids. On the other hand, we observed no decreases of either the saturated or unsaturated (mono- and polyunsaturated) fatty acids in rats exposed to ozone + DEP (Figure 3). These results suggested that the loss of mitochondrial PUFA might involve phospholipase-mediated or nonenzymatic hydrolysis (deacylation) and/or peroxidation.

### Acute ozone- and DEP-induced effects in the lung and aorta

As expected, we observed no significant changes in lung inflammation or injury markers in rats exposed to 0.5 ppm ozone or DEP as assessed in BALF, but 1.0 ppm ozone increased neutrophils, which was also reflected in total cell increase [see Supplemental Material, Figure 5 (doi:10.1289/ehp.1002386)]. Similarly BALF protein and albumin also increased after exposure to 1.0 ppm ozone. We found no noticeable changes in systemic levels of white or red blood cells and adiponectin (see Supplemental Material, Table 5). DEP, but not ozone, at 0.5 ppm increased *MIP-2*, *tPA*, *LOX-1*, and *RAGE* mRNA in the lung (see Supplemental Material, Figure 6). In the lung, 0.5 ppm ozone increased *PAI-1* expression, and at 1.0 ppm increased *HO-1* expression. The changes observed in the aorta were less remarkable after acute exposure compared with 16-week exposure. We observed no changes in aortic mRNA biomarkers of thrombosis, inflammation, or proteolysis in any exposure conditions (see Supplemental Material, Figure 7). The only significant increases were observed in *ET-1* mRNA after exposure to 1.0 ppm ozone or DEP, and in *ETR-B* after 1.0 ppm ozone. Because aortic *LOX-1* and *HMGB-1* mRNA in rats exposed to 0.5 ppm ozone tended to be lower than in air controls, the comparison between 0.5 ppm ozone and DEP yielded a statistically significant difference. However, no significant changes were discernible in *LOX-1* and *HMGB-1* relative to air controls in ozone- or DEP-exposed rats (see Supplemental Material, Figure 7).

## Discussion

Credible explanation of how inhaled pollutants, particulate or gas, might induce systemic vascular and cardiac molecular alterations remains an area of intense research in the field of environmental cardiology. In this study, we asked if biologically significant and consistent vascular and cardiac effects can be observed in rats inhaling *a*) DEP, which increases vasoconstriction in humans (Peretz et al. 2008) and accelerates atherosclerosis in apolipoprotein E–null (ApoE^−/−^) mice (Campen et al. 2010; Peretz et al. 2008); *b*) ozone, which produces marked cardiophysiological effects in rats (Uchiyama et al. 1986); or *c*) ozone + DEP. We focused on the biomarkers related to inflammation, oxidative stress, microvascular thrombosis, vasoconstriction, and proteolysis in three tissues: lung, heart, and thoracic aorta. In the episodic 16-week exposure scenario, mRNA expression of any of the biomarkers was not affected in the heart either by ozone, DEP, or ozone + DEP. Small increases were noted in only a few markers of inflammation and thrombosis in the lung after ozone or DEP exposure. However, marked up-regulation was observed in aortas from rats exposed to ozone or DEP alone, but not ozone + DEP (*HO-1, tPA, PAI-1, vWf, Thbd, ET-1, ETR-A, eNOS, MMP-2*, and *TIMP-2*). Changes induced by acute exposure were less remarkable. Because binding of circulating oxidatively modified protein and lipid adducts to RAGE and LOX-1 on the endothelial surface induces expression of these biomarkers and mediates vascular pathogenesis of diabetes and atherosclerosis (Navarra et al. 2009; Yan et al. 2009), we postulated that these receptors will be up-regulated in the aorta as a result of increased oxidation by-products generated in response to ozone or DEP exposure. *LOX-1* but not *RAGE* mRNA and protein levels were up-regulated in rats exposed to ozone, whereas *RAGE* and *HMGB-1* mRNA increased only in rats exposed to ozone + DEP. In our previous study of 4-week inhalation to whole diesel exhaust (Gottipolu et al. 2009), we noted no changes of inflammatory and prothrombosis biomarkers in the heart, but we observed inhibition of myocardial mitochondrial aconitase activity and suppression of compensatory genes; therefore, we postulated that ozone and DEP exposure might cause cardiac mitochondrial membrane PUFA to be altered because of oxidative stress. In the present study, we observed that several PUFA in cardiac mitochondria decreased after weekly inhalation exposure to ozone or DEP, suggesting oxidative modifications. These effects of ozone or DEP alone were less remarkable in rats exposed to ozone + DEP. Thus, we provide the evidence that episodic 16-week, but not acute, exposure of rats to ozone or DEP alone induces common and pollutant-specific aortic and cardiac molecular alterations. We postulate that oxidatively modified lipid and protein mediators produced within the lung and heart might promote vascular pathology through LOX-1 and/or RAGE signaling in a pollutant-specific manner.

The changes we observed in the aorta of healthy rats are interesting in light of the reported increases in plaque size and oxidative stress in ApoE^−/−^ mice after long-term exposure to DEP or concentrated ambient PM (Campen et al. 2010; Sun et al. 2005). Increased superoxide generation and mRNA expression of NADPH oxidase, and iNOS (inducible nitric oxide synthase) after subchronic PM exposure have been proposed to be mediated via GTP signaling (Ying et al. 2009). Increased activation of *ras* homolog gene family, member A (RhoA)/Rho-kinase signaling has been postulated to mediate hypertension in mice subchronically exposed to ambient PM (Ying et al. 2009). More recently, subchronic ozone exposure has also been shown to increase oxidative modifications in the mouse aorta (Chuang et al. 2009). Although the evidence suggests that oxidative stress might be involved in vascular effects of air pollutants, individual studies have been performed with limited tissue targets, one pollutant, or only a few biomarkers. Thus, where oxidative stress is generated and how it affects vasculature versus myocardial tissue remains unclear.

Upon binding of oxidatively modified lipids, vascular LOX-1 receptors signal downstream activation of mitogen-activated protein kinases (MAPKs) and NFκB, together with increased MMPs, endothelin-1, and inhibition of protein kinase B (PKB/Akt), leading to apoptosis (Navarra et al. 2009). Activation of NFκB causes gene expression of inflammatory proteins, MMPs, PAI-1, and TF and increased foam cell accumulation within the vessel. Similarly, RAGE activation also mediates downstream signaling through NFκB, which is linked to endothelial dysfunction (Navarra et al. 2009). HMGB-1, recently known as the “late” proinflammatory mediator of systemic inflammation, has been shown to activate MAP kinase and NFκB signaling through RAGE (Sims et al. 2010). Oxidation of proteins and lipids is likely within the lung lining and also at extrapulmonary sites such as heart after ozone or DEP exposure. Ozone oxidizes a number of lipid moieties of surfactant, especially phosphatidylethanolamine, and generates reactive lipid mediators (Wynalda and Murphy 2010). Ozone also can oxidize pulmonary proteins (Uhlson et al. 2002). Further, pulmonary inflammation might also lead secondarily to oxidation of lipids and proteins within the lung lining and epithelium. We postulate that, if unrepaired, these oxidation by-products escape into the circulation and mediate endothelial signaling through LOX-1 and RAGE. Data from the present study show that *LOX-1* gene and protein were both increased in the rats exposed to ozone and that *RAGE* mRNA was increased in the case of ozone + DEP, and these were associated with up-regulation of biomarkers of oxidative stress, thrombosis, vasoconstriction, and increased proteolytic activity. DEP-induced up-regulation of these biomarkers in the aorta might be mediated through HMGB1 and possibly RAGE signaling. Although both ozone and DEP caused similar vascular effects in a few biomarkers in the 16-week study, critical differences existed for aortic *LOX-1* mRNA and protein induction with different exposures. Identification of the precise mechanisms and oxidation by-products in the circulation is needed to further support these hypotheses.

The lack of effect of DEP or ozone on cardiac pathology or gene expression of biomarkers examined in the present study supports results of our previous study (Gottipolu et al. 2009) in which 1-month exposure to inhaled diesel exhaust showed no induction of these same biomarkers in the heart. However, marked repression of cardiac genes related to compensatory responses and structural components, as well as mitochondrial aconitase activity, in that study prompted us to examine the effect of ozone and DEP on composition of fatty acid phospholipids as an index of oxidative stress in the myocardial mitochondria. PUFA concentrations were decreased in myocardial mitochondria after exposure to ozone and DEP alone, likely due to phospholipase-mediated or nonenzymatic oxidation (Jin and Zhou 2009; O’Connor Butler et al. 2010). The loss of phospholipids might impair contractile response of the myocardium. These results show that, in the given experiment, molecular alterations in the myocardium are distinct from those in the systemic vascular tissues, and might reflect the nature of tissue and the functional properties of each cell type. Ultimately, after > 16-week exposure, cardiac tissues might also be prone to inflammatory pathology.

We observed effects of ozone and DEP on the aorta only after 16-week episodic exposure. The small effect on ET-1 and the tendency toward an increase in MMPs after acute exposure may suggest that these effects progressively increased over the course of exposure. The role of HMGB1, together with changes in blood hemoglobin, lymphocytes, and platelets, may be critical in progression of vascular effects over a long period and needs to be examined further. The episodic nature of exposure could have resulted in a pattern of reexposure when adaptive responses had already subsided at each successive week. It is noteworthy that marked vascular effects were apparent without substantial lung inflammation.

Although the ozone and DEP exposure concentrations we used are higher than would be expected in urban areas, they may relate to pollutant deposition during exercise in humans exposed to high ozone and PM or during extreme episodic spikes of high ambient levels, especially in developing countries. One unexpected outcome of the present study was the generalized diminished response of ozone + DEP relative to individual pollutant exposure in the 16-week study. Human exposure to gas and particulate air pollution occurs as a mixture, but very few studies have examined biological effects of mixtures, although human studies have shown additive effects of DEP and ozone (Bosson et al. 2008). In the present study, efforts were made to minimize interaction time between ozone and DEP and to maintain steady target ozone concentration at nose-only port. Nonetheless, the ozone concentrations at the nose-only port was slightly lower than the target ozone concentration in the ozone + DEP chamber (0.37 ppm as opposed to 0.4 ppm in ozone-only chamber), supporting the possibility of some degree of chemical reaction prior to interacting with biological molecules in the airway lining. Ozone + DEP could chemically interact in the air before the encounter with lung lining fluid (Jeong and Park 2008) or independently affect biological processes with diminished ultimate response (Thomson et al. 2005). Deactivation of reactive organic polycyclic aromatic hydrocarbons on the DEP has been shown to deplete ozone (Poschl 2002). This interactive aspect of ozone and DEP becomes highly relevant because these major air pollution components might undergo chemical modifications during atmospheric aging.

## Conclusion

Our data show that weekly episodic exposure to ozone (which does not translocate systemically) or DEP alone—but not the combination (ozone + DEP)—causes marked mRNA up-regulation of biomarkers of oxidative stress, protease/antiprotease balance, microvascular thrombosis, and vasoconstriction in the aorta. These effects were not evident in the heart. We also observed that cardiac mitochondrial phospholipid fatty acids decrease after exposure to ozone or DEP alone but not ozone + DEP, likely resulting in oxidative modifications. Biomarkers of vascular pathogenesis and associated increases in vascular *LOX-1* and/or *RAGE* expression might be mediated through circulating oxidation by-products of lipids and proteins.

## Correction

The name of Narasimham L. Parinandi was misspelled in the original manuscript published online. It has been corrected here.
